# Identification a novel de novo* RUNX2* frameshift mutation associated with cleidocranial dysplasia

**DOI:** 10.1007/s13258-022-01229-w

**Published:** 2022-03-02

**Authors:** Lei Gong, Bekzod Odilov, Feng Han, Fuqiang Liu, Yujing Sun, Ningxin Zhang, Xiaolin Zuo, Jiaojiao Yang, Shouyu Wang, Xinguo Hou, Jianmin Ren

**Affiliations:** 1 Department of Endocrinology, Qilu Hospital, Cheeloo College of Medicine, Shandong University, Jinan, 250012 China; 2 Institute of Endocrine and Metabolic Diseases of Shandong University, Jinan, 250012 China; 3 Key Laboratory of Endocrine and Metabolic Diseases, Shandong Province Medicine & Health, Jinan, 250012 China; 4 Jinan Clinical Research Center for Endocrine and Metabolic Diseases, Jinan, 250012 China; 5 Department of Endocrinology, Zhangqiu District People’s Hospital, Jinan, 250200 China

**Keywords:** *RUNX2*, Novel mutation, Cleidocranial dysplasia, Genetic disorder

## Abstract

**Background:**

Cleidocranial dysplasia (CCD) is a rare genetic disorder affecting bone and cartilage development. Clinical features of CCD comprise short stature, delayed ossification of craniofacial structures with numerous Wormian bones, underdeveloped or aplastic clavicles and multiple dental anomalies. Several studies have revealed that CCD development is strongly linked with different mutations in runt-related transcription factor 2 (*RUNX2*) gene.

**Objective:**

Identification and functional characterization of *RUNX2* mutation associated with CCD.

**Methods:**

We performed genetic testing of a patient with CCD using whole exome sequencing and found a novel *RUNX2* frameshift mutation: c.1550delT in a sporadic case. We also compared the functional activity of the mutant and wild-type *RUNX2* through immunofluorescence microscopy and osteocalcin promoter luciferase assay.

**Results:**

We found a novel *RUNX2* frameshift mutation, c.1550delT (p.Trp518Glyfs*60). Both mutant *RUNX2* and wild-type *RUNX2* protein were similarly confined in the nuclei. The novel mutation caused abrogative transactivation activity of *RUNX2* on osteocalcin promoter.

**Conclusions:**

We explored a novel *RUNX2* deletion/frameshift mutation in a sporadic CCD patient. This finding suggests that the VWRPY domain may play a key role in *RUNX2* transactivation ability.

## Introduction

Cleidocranial dysplasia (CCD), also referred as a Scheuthauer syndrome, is a rare autosomal dominantly inherited disorder which is represented by various skeletal abnormalities (Lotlikar et al. 2018). The global CCD occurrence rate is usually one in every millions of newborns without gender preponderance (Offiah et al. 2019). Clinical features of CCD is diverse and involves short stature, delayed ossification of craniofacial structures with numerous Wormian bones, underdeveloped or aplastic clavicles, multiple dental defects such as teeth hypoplasia, delayed primary teeth exfoliation, prolonged eruption of permanent teeth and malocclusion (Konishi et al. 2019). Other skeletal abnormalities, such as hypoplastic iliac wings, distal phalanx dysplasia, knock-knees and malformations of spine can be also observed (Farrow et al. 2018). The CCD can be diagnosed prenatally, from early childhood till late adolescence and diagnostic tools ranges from ultrasound investigations (in prenatal period) to panoramic radiography, although early diagnosis is considered as aTkey problem in CCD management (Zeng et al. 2018).

Several studies have revealed that CCD development is strongly linked with different mutations affecting runt-related transcription factor 2 (*RUNX2*) gene (Jaruga et al. 2016; Xuan et al. 2008), which is a transcription factor involved in osteoblastic differentiation and skeletal morphogenesis. Human *RUNX2* gene consisting 8 coding exons, is located in chromosome 6p21 (Levanon et al. 1994). *RUNX2* gene comprises Q/A domain on N-terminal part, RUNT domain which is crucial for DNA-binding to a specialized motif and heterodimerization with core-binding factor subunit beta (CBFβ) (Zhang et al. 2017), as well as proline/serine/threonine-rich (PST) domain on its C-terminus (Yoshida et al. 2002). The core-binding factor subunit alpha-1 (CBFA1) protein, that *RUNX2* gene encodes is expressed densely in skeletal structures and considered as a key transcription factor for numerous stages of osteogenesis (Sun et al. 2016). Noticeably, mice with targeted *RUNX2* disruption have bone formation failure owing to osteoblast deficiency (Zhong et al. 2016). Moreover, it also has been suggested that *RUNX2* represents crucial role in tooth development, and its transcriptional failure could promote dental lamina excess activation leading to supernumerary teeth with consequent effect on permanent teeth (Wen et al. 2020). Till recent years, almost 194 causative mutations in *RUNX2* have been identified, and this number is still rising (Otto et al. 2002). However, *RUNX2* mutations can be found only in two-third of patients with CCD, and 30%–40% of cases are triggered by novel mutations (Hordyjewska-Kowalczyk et al. 2019).

Although we have known more about the clinical and functional characteristics of *RUNX2* because of recent studies, there are still some potential unknown factors that urge further exploration. *RUNX2*, as the key contributory gene in CCD, is conceivable to be a therapeutic target for CDD. In current study, we explored a novel *RUNX2* deletion mutation: c.1550delT (p.Trp518Glyfs*60) in a sporadic case and also showed its clinical features, possible pathogenesis and functional characteristics.

## Materials and methods

### Subjects

The subjects were examined in the Department of Endocrinology, Qilu Hospital of Shandong University, China. The peripheral blood samples were obtained for genetic testing from all participants, including 15 year-old boy with suspected CCD and his unaffected parents. We also carried out clinical and radiological examinations on patient, and collected his medical history.Current study was accepted by the ethics committee of QiLu hospital of Shandong University (ethical approval number KYLL-2019-2-111). All subjects in our study signed consent form voluntarily with the review of the ethical committee. The study methods were performed according to the ethical committee accepted guidelines.

### Mutation analysis

The whole-exome sequencing (WES) was carried out on DNA from venous blood sample. Fragmentation of the genomic DNA, paired-end adaptor ligation, amplification and purification were implemented, and the all human exons together with 50 bp bases in their adjacent introns were captured by xGen® Exome Research Panel. The DNA library was performed post-capture amplification and purifying, and then arrayed by the Illumina HiSeq sequencing platform. All test and sequence analysis were supplied by the Beijing Fujun Gene Biotechnology Co., Ltd (Beijing, China).

### In silico assays for a Runx2 frameshift

Using phyer2 (http://www.sbg.bio.ic.ac.uk/phyre2/html/page.cgi?id=index), the WT-*RUNX2* and W518Gfs-*RUNX2* structural conformation were comprehensively analyzed and predicted using the threading method and the heavy-head prediction method, respectively, and the WT-*RUNX2* and W518Gfs-*RUNX2* structural models were established as the reference instruction (Kelley et al. 2015). Additionally, SAVE5.0 3D Structure Viewer (https://saves.mbi.ucla.edu/) to visualize 3D structure has been used.

### Cell culture and transfection

Human embryonic kidney 293 (HEK293) cells were cultured in Dulbecco’s modified Eagle’s medium basic (DMEM basic, Gbico, Grand Island, NY, USA, cat: C11995500BT) supplemented with 10% fetal bovine serum (FBS, Gbico, Grand Island, NY, USA, cat: 10,099,141), penicillin (100 IU/mL), and streptomycin (100 μg/mL) as formerly detailed (Hu et al. 2014; Wang et al. 2014). Cells transfection with the plasmids carrying needed genes by using Lipofectamine™ 2000 (Invitrogen, Carlsbad, CA, USA) was performed to investigate protein expression and other related studies.

### Western blotting analysis

We used the human embryonic kidney (HEK) 293 cells to study the WT- *RUNX2* and W518Gfs-*RUNX2* expression as previously described. For western blotting analysis, the cells were seeded in 10 cm^2^ plates. One day later, the HEK293 cells were transfected with pcDNA3.1-GFP, pcDNA3.1-WT-*RUNX2*-GFP and pcDNA3.1 W518Gfs-*RUNX2*-GFP*.* After 48 h transfection, the cells were cultured and subjected to SDS–PAGE. Western blotting was performed with a rabbit polyclonal anti-GFP antibody (1:1000, proteintech, Wuhan, Hubei, China, cat: 50,430-2-AP) and a horse radish peroxidase-conjugated goat anti-rabbit IgG polyclonal antibody (1:10,000, Zhongshan Golden Bridge, Beijing, China, Cat: ZB-2301) as a second antibody. Signals were detected using a chemiluminescence kit (Millipore, California, USA, Cat: WBKLS0050). Each experiment was repeated three times.

### Luciferase reporter assays

Previously, seeded the HEK-293 cells into 96-well culture plate 24 h before this assay, 10,000/well, 200 ul cell suspension, culture at 37 ℃, 5% CO_2_ (Sun et al. 2020). On the second day, pRL-TK and pGL3-basic-osteocalcin promoter plasmid has been added to each group, and each group were also transfected separately with different concentrations of pcDNA3.1, pcDNA3.1-WT-*RUNX2*, pcDNA3.1-W518Gfs-*RUNX2* (0 ug, 1 ug, 2 ug). After 48 h, the follow-up dual-luciferase detection operation was performed. For details, we followed the dual-luciferase reporter assay system and analyzed the results.

### Statistics

Data were presented as the mean ± standard error of mean (SEM) from at least three independent experiments. Statistical comparisons were carried out using paired t-test, ANOVA or Bonferroni test with GraphPad Prism 6.0 (GraphPad Software, San Diego, California, USA). The protein bands from the western blot were quantified applying Image J software (National Institutes of Health, Bethesda, Maryland, USA). Each experiment was repeated at least in triplicate. Statistically significant differences were considered at p value < 0.05.

## Results

### Clinical manifestation

**Fig. 1:**
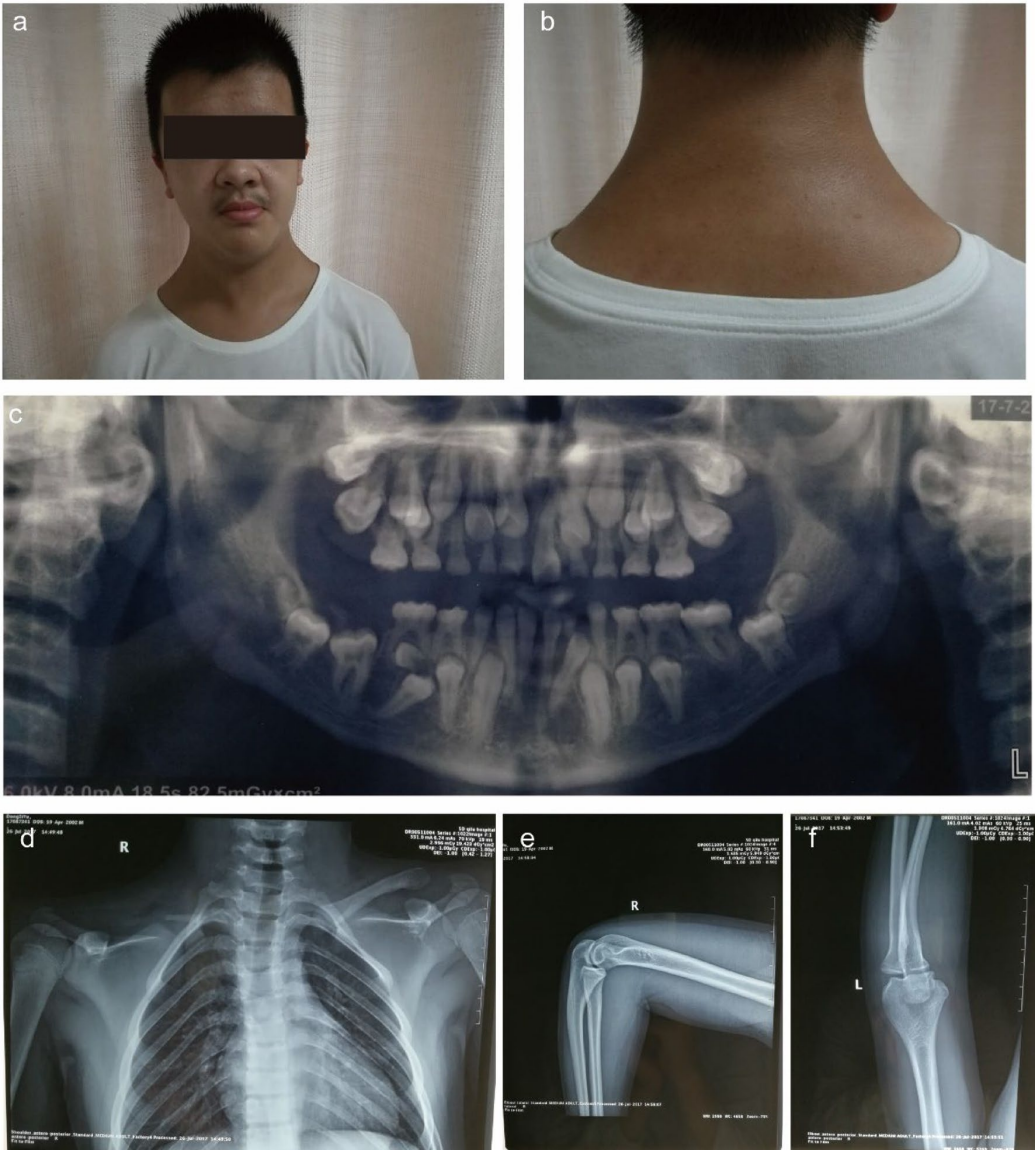
Typical and radiological findings in the CCD patient. **a** Frontal facial view of patient representing midline depression of forehead and bilateral hypoplastic clavicles. **b** Hypoplasia of the clavicles abnormal facility in the opposing shoulders. **c** Panoramic radiography revealed primary teeth retention, numerous impacted permanent teeth in both maxilla and mandible. **d** Chest X-ray showed bilateral hypoplastic clavicles hypoplasia of iliac bones, wide symphysis pubis with a bell-shaped thoracic cavity and scoliosis. **e**, **f** No abnormalities were seen in the bones of the bilateral elbow joints, and the epiphyseal line showed closure

Among three participants of our study, diagnosis of CCD was confirmed only in 15-year old male proband. His past history and physical examinations has differed noticeably from his parents. On extraoral examination, typical features of CCD, such as midline depression of upper forehead, narrow and dropped shoulders and short stature were observed. His photographs were obtained and he was referred to radiographic investigations (Fig. 1).

### Oral manifestations and radiographic features

His panoramic radiograph showed mixed dentition with delayed exfoliation of deciduous teeth, retained and impacted permanent teeth. Aplasia of lateral two-thirds of right and one-third of left clavicles, cone shaped thorax, scoliosis and deformed ribs were found during chest radiography (Fig. 1).

### Sequencing results and biochemical characterization of *RUNX2* (c.1550delT) gene mutation

**Fig. 2:**
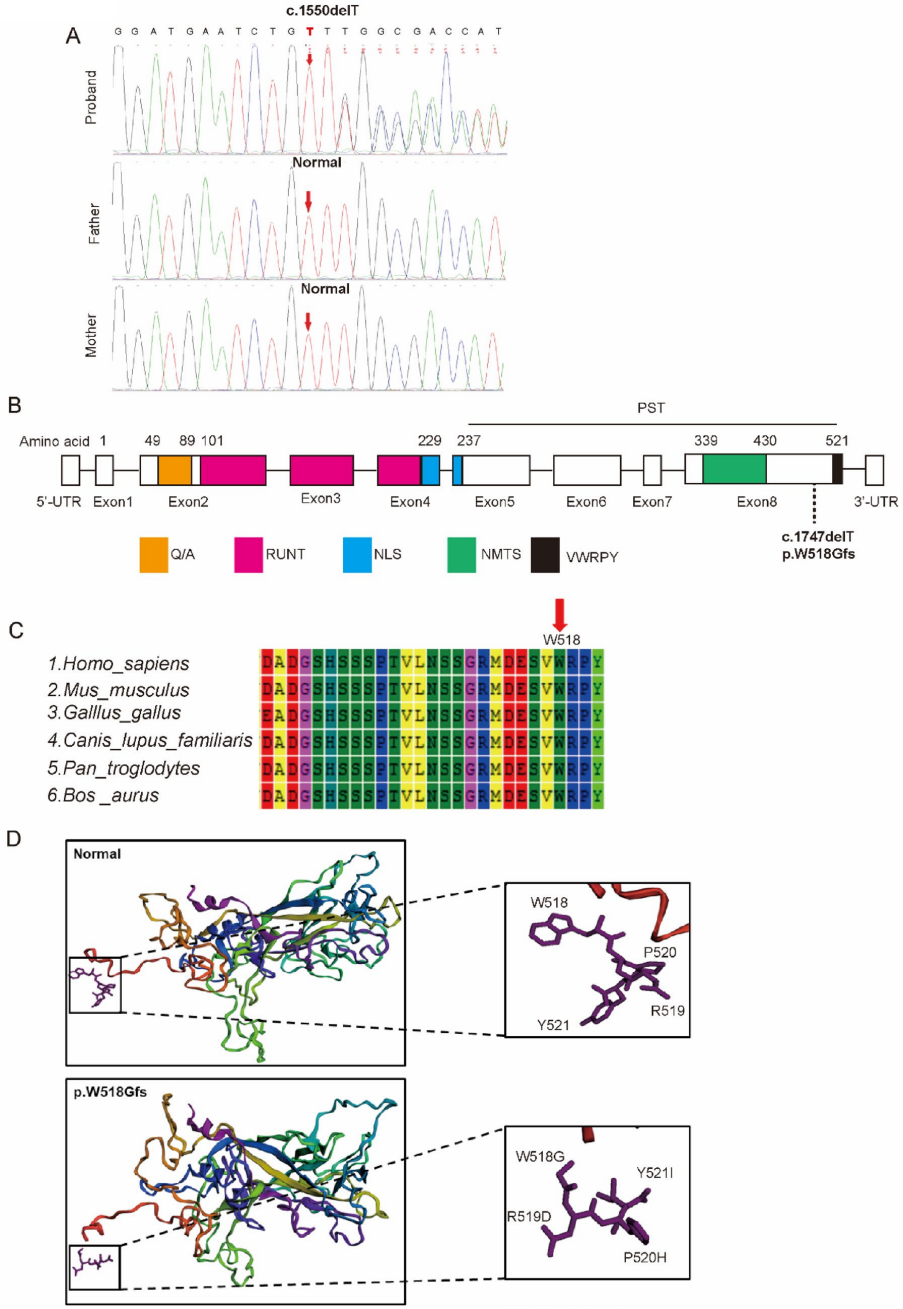
Partial sequence diagram of *RUNX2* and biochemical characterization of the *RUNX2* (c.1061G > T) variant. **A** Partial sequence diagram of *RUNX2*. A heterozygous c.1550delT transition mutation is shown using an arrow (GenBank accession number: NM_001024630.4). This frameshift mutation resulted in changes in amino acid synthesis starting from amino acid Trp 518 (p.Trp518Glyfs). **B** *RUNX2* structural domains. Mutations at the protein level are indicated below the PST domain. **C** Cross-species conservation of Trp518-*RUNX2*. **D** Protein structure prediction of the *RUNX2* (WT and Trp518Glyfs). *RUNX2* WT protein sequence is ^518^WRPY^521^ and the *RUNX2* (c.1550delT) mutant sequence is ^518^GDHI^521^

Sequencing analysis was implemented in the coding region of *RUNX2* gene. *RUNX2* (c.1550delT) mutant was detected in the proband, but not in his parents (Fig. 2A). The c.1550delT mutation was clustered in the terminal VWRPY of highly conserved PST domain (Fig. 2B–C). It is predicted that mutations at this site may change the protein activity (Fig. 2D).

### Functional characterizations of *RUNX2* (c.1550delT) gene mutation

**Fig. 3:**
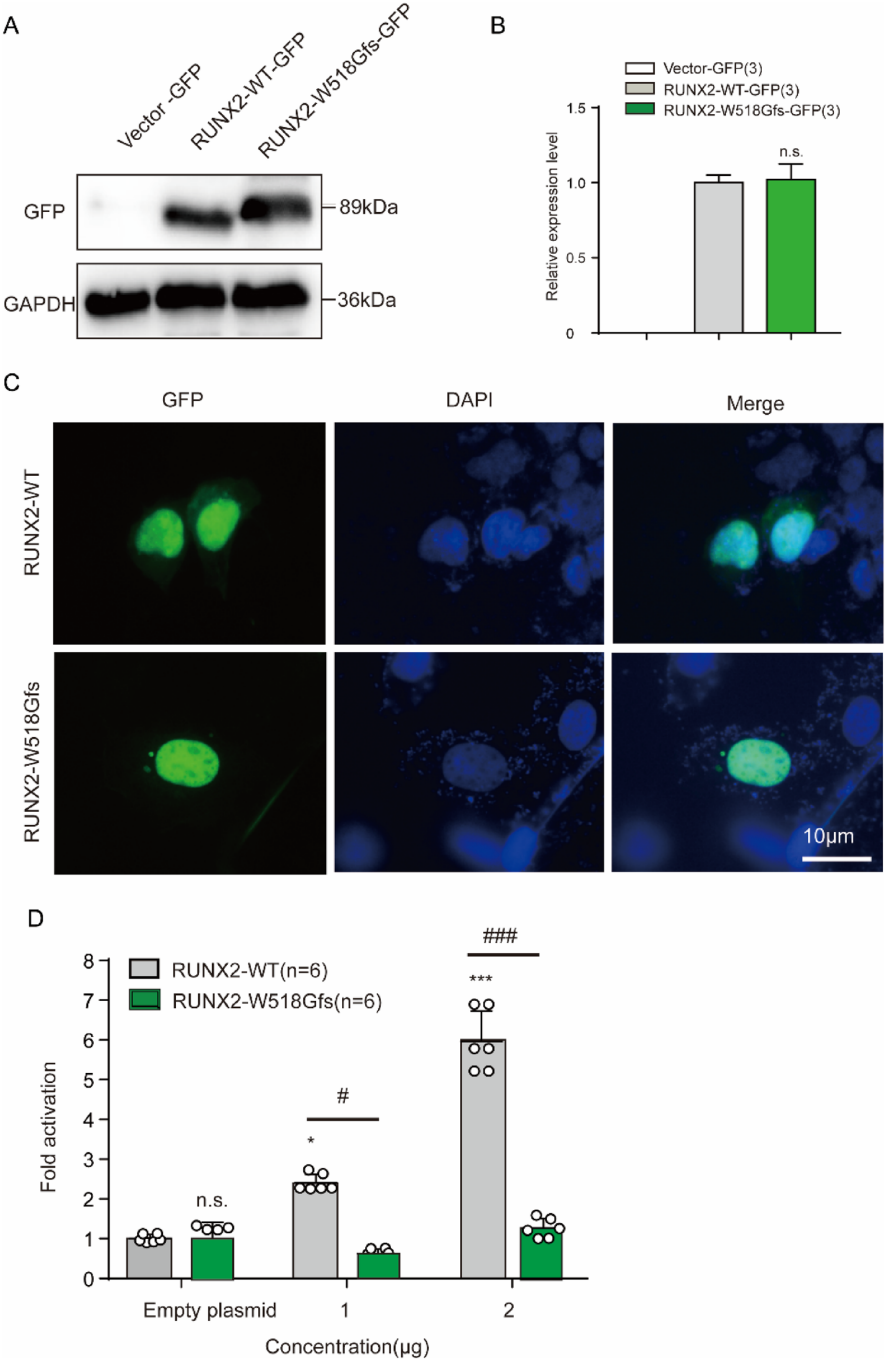
Functional characterization of the *RUNX2* (c.1061G > T) variant. **A** Protein expression of *RUNX2* (WT and Trp518Glyfs). **B** The histogram of the *RUNX2* protein expression level analysis. n.s., denotes *RUNX2* Trp518Glyfs compare with the empty denotes *p* > 0.05. **C** Nuclear localization of WT and mutant *RUNX2*. **D** Luciferase results of HEK293 cells were transfected with each *RUNX2* expression vector (WT and Trp518Glyfs). *, denotes *RUNX2* WT plasmid compare with the empty plasmid, *p* < 0.05; #, denotes *RUNX2* Trp518Glyfs compare with *RUNX2* WT, *p* < 0.05, *n* = 6

Western blot analysis confirmed that *RUNX2* (c.1550delT) mutant gene did not cause reduction in *RUNX2* protein expression level (Fig. 3A, B). Some studies have illustrated that *RUNX2* is predominantly located in the cell nucleus and little perinucleolar region (Javed et al. 2000; Young et al. 2007). In order to verify the *RUNX2* mutation nuclear localization, we transfected both pGFP-*RUNX2* and pGFP-*RUNX2*-Trp518Glyfs plasmids into HEK293T cells. The subcellular localization of the *RUNX2* and the Trp518Glyfs mutation was observed by in situ immunofluorescence microscopy (Young et al. 2007). Both *RUNX2* mutation and wild-type *RUNX2* accumulated in the nuclei of HEK293T cells (Fig. 3C). This denoted that the subcellular compartmentation of the *RUNX2* mutation was not affected. It has been reported in previous studies that *RUNX2* is responsible for the transactivation of the osteoblast-specific osteocalcin gene in osseous cells (Zaidi et al. 2001). Luciferase assays demonstrated the *RUNX2* transactivation activity and the transcriptional regulation of the osteocalcin promoter. The *RUNX2* (c.1550delT) variant induced the osteocalcin promoter activity lower than *RUNX2*-WT (Fig. 3D).

## Discussion

Human *RUNX2* gene comprises Q/A domain on N-terminal part, RUNT domain, as well as PST domain on its C-terminus. More noticeably, RUNT domain include NLS on its C-terminus, and this part has been reported to be important for the protein nuclear transportation (Ryoo et al. 2010). It also has been found that PST domain, to be more specific, its nuclear matrix targeting sequence (NMTS)-associated subnuclear foci is engaged in both activation of downstream factors such as osteocalcin gene and subnuclear localization of *RUNX2* (Zaidi et al. 2001).Similarly, Q/A domain also participates in transactivation activity of *RUNX2* target genes (Kauffenstein et al. 2016).

In our study, we found a novel *RUNX2* (c.1550delT) mutation,causing frameshift starting from 518 codon, eventually led to aberrant VWRPY domain in C-terminus. The protein sequence of *RUNX2* WT ^518^WRPY^521^ is replaced by ^518^GDHIEIPQQWPSGIWGPHPTRINIYIYRESAYICISISYLQSAYFLEDFSFTHSVMILQP^577^. The local spatial structure of the protein is changed because the local secondary structure of the protein changes from alpha helix to random coil. Stop codon of *RUNX2* WT is TGA at gene site 1566. Stop codon of c.1550delT is TAA at gene site 1734. Considerably, neither *RUNX2* protein expression, nor its subcellular distribution was impaired in current study, which might be explained by intact RUNT domain. Several studies have proposed that osteocalcin promoter activation is VWRPY-dependent (Qin et al. 2017). In the present study, abnormal VWRPY domain led to aberrant downstream activation of osteocalcin promoter that further supported previous findings. Zaidi et al. suggested that VWRPY is not crucial for *RUNX2* nuclear retention (Zaidi et al. 2001). Since we have not found defective nuclear retention of *RUNX2*, our findings approved the point of Zaidi et al*.*

Currently, more than 48 phenotypic characteristics of CCD have been registered by OMIM (Qin et al. 2017). Although, numerous previous studies aiming to reveal genotype–phenotype association have been performed, controversy related to this point is still exist (Quack et al. 1999; Zhang et al. 2010). Additionally, some studies failed to find association between *RUNX2* mutation and the severity of CCD (Lou et al. 2009). Moreover, threshold level of *RUNX2* mutation that initiates CCD remains unidentified (Xu et al. 2017). Although CCD is considered as autosomal dominant disease, as stated in recent studies, it can be present in a sporadic pattern almost in 30% of cases (Huang et al. 2013). Notably, the genetic testing for possible mutations revealed that both of patient’s parents had normal *RUNX2* in our study, suggesting that mutation occurred de novo. Although VWRPY is not hotspot for mutations and aberrant VWRPY did not lead to impaired *RUNX2* protein synthesis, subcellular distribution in present research, it precipitated CCD with classical phenotype. This might be explained by possible influence of other determinants on CCD phenotype.

To conclude, we explored a novel *RUNX2* deletion/frameshift mutation in a sporadic CCD patient. This finding emphasizes on crucial role of VWRPY domain in *RUNX2* transactivation ability. Further studies are awaited to explore more *RUNX2* mutations for revealing potential attributors as well as genotype–phenotype association.

## Data Availability

All data generated or analysed during this study are included in this published article.
